# Intimate Partner Violence and Mental Distress, Post-Traumatic Stress Symptoms and Life Satisfaction in Colombian Women

**DOI:** 10.3390/bs14100940

**Published:** 2024-10-14

**Authors:** Janneth E. Molina, M. Pilar Matud

**Affiliations:** 1 Faculty of Psychology, Universidad Católica de Colombia, Av. Caracas #46-72, Bogotá 11112, Cundinamarca, Colombia; 2 Department of Clinical Psychology, Psychobiology, and Methodology, Universidad de La Laguna, 38200 San Cristobal de La Laguna, Spain

**Keywords:** intimate partner violence, women, mental distress, post-traumatic stress symptoms, life satisfaction, self-esteem, social support, Colombia

## Abstract

Intimate partner violence (IPV) against women is a major global public health and human rights issue, with significant consequences for women’s health and well-being. This study aimed to determine the relevance of IPV on women’s mental distress, post-traumatic stress symptoms and life satisfaction, and to examine whether age, education, socioeconomic status, social support and self-esteem are associated with mental health and well-being. The sample consisted of 255 women aged 18 to 71 living in Colombia who were assessed using six self-reported questionnaires. Hierarchical multiple regression analyses showed that more IPV was associated with women’s increased mental distress, post-traumatic stress symptoms and decreased life satisfaction. Although self-esteem and, to a lesser extent, social support were important predictors of women’s mental distress and well-being, psychological IPV was the main predictor of women’s post-traumatic stress symptoms, followed by lower self-esteem and younger age. The most important predictor of women’s mental distress was lower self-esteem, followed by more psychological IPV and lower social support. The main predictor of women’s life satisfaction was high self-esteem, followed by high social support, less psychological IPV and higher age and education. It is concluded that IPV, especially psychological IPV, is a serious threat to women’s mental health and well-being, while high self-esteem and, to a lesser extent, high social support are associated with better mental health and well-being.

## 1. Introduction

Intimate partner violence (IPV) against women is a serious public health problem and human rights violation worldwide [1,2,3,4]. IPV applies to violence inflicted by both current and former spouses and partners. It is defined as “behaviour within an intimate relationship that causes physical, sexual or psychological harm, including acts of physical aggression, sexual coercion, psychological abuse and controlling behaviours” [5] (p. 11). According to the World Health Organization (WHO) [3], physical, sexual or psychological violence perpetrated by a male intimate partner is the most common form of violence against women globally. The global lifetime prevalence of physical and/or sexual violence by a current or former intimate partner among women aged 15–49 years is estimated to be 27% and the prevalence in the past year is estimated to be 13%. However, there are important regional differences, with higher rates in low-income countries compared with high-income countries [2,3]. Regions with lower prevalence include Australia and New Zealand, where lifetime prevalence is 23% and past-year prevalence is 3%, and southern Europe, where lifetime prevalence is 16% and past-year prevalence is 4% [3]. In the Americas region, the lifetime prevalence of physical or sexual violence by a current or former partner among women aged 15–49 years is 25% and the past-year prevalence is 7%, although there is wide variation across countries [3,6]. Colombia is among the countries with the highest rates of physical or sexual IPV, with a lifetime rate of 30% and a past-year rate of 12% [3].

Although many estimates of global rates of IPV against women do not include an analysis of the prevalence of psychological violence, perhaps because there is little agreement on how to conceptualize or measure psychological IPV [7,8], there is evidence that it is common [7,9,10,11]. A recent systematic review and meta-analysis [11] of 201 studies involving 250,599 women, mostly from high-income countries, published between 2012 and 2020 found that lifetime psychological violence was the most common form of IPV.

IPV occurs across locations, social strata and cultural backgrounds [12]. However, its estimated prevalence varies by sociodemographic characteristics [12,13,14,15,16,17,18,19,20], with lower prevalence among older women [2,13,14,15,16,19]. Although it is recognized that rates of IPV are highest among women of reproductive age, IPV is also prevalent among women over the age of 50 [13,14,15,16,21,22], though it has been suggested that the nature and dynamics of IPV may change as women get older [14,15]. Other factors that have been associated with a higher prevalence of IPV include low educational attainment [13,17,18,19] and low socioeconomic status [13,19,20]. It has been argued that IPV is driven by structural factors such as poverty, including low educational attainment and food insecurity, gender inequality and the normative use of violence in interpersonal relationships [23]. There is evidence that its prevalence is also associated with contextual factors at the country level, with lower rates in countries and regions with higher incomes, higher rates of secondary or higher education among women and greater economic equality between women and men [1].

IPV against women has significant short, medium and long-term consequences for the physical and mental health and well-being of women, children and families. It also has a major social and economic impact on societies and nations [3]. The effects of IPV persist and continue to affect women into old age, even after the violence has ended. There is evidence that older women survivors of IPV have poorer mental health, higher risk of depression and greater vulnerability to abuse [24]. Mental, physical, sexual and reproductive health problems and death (by homicide and suicide) are associated with IPV against women [9,12,16,18,19,21,22,23,24,25,26,27,28,29,30]. In addition, for young women, IPV also affects their educational well-being, with increased absenteeism, lower academic performance and higher dropout rates. All of these have significant long-term consequences [31]. Although many mental health problems are reported, depression [1,9,11,28,32,33,34,35,36], post-traumatic stress symptoms [11,30,33,35,37,38] and mental distress [30,32,36,39,40,41,42] are among the most reported. While there is considerable research on the impact of IPV on women’s mental health, little research has focused on examining the impact of IPV victimization on women’s well-being. Traditionally, health has been thought of as the absence of illness or disability [43], but the dual-factor model of mental health suggests that distress and well-being are two related but distinct constructs [43,44]. Therefore, in the present work, we will not only analyze women’s mental symptomatology, but also their life satisfaction, which is the cognitive component of subjective well-being [45].

The main purpose of this study is to advance our understanding of the mental health and subjective well-being of women exposed to IPV. Specifically, we examine how physical, psychological and sexual IPV predicts women’s mental distress, post-traumatic stress symptoms and life satisfaction. In addition, we seek to understand the importance of age and socioeconomic and educational levels, as well as self-esteem and social support, on mental distress, post-traumatic stress symptoms and life satisfaction among women who experienced IPV.

The study was conducted in the Latin American country of Colombia, a region that has high rates of IPV against women but is generally underrepresented in the IPV-related literature [46,47]. Research on IPV among older women in this region is also scarce [48]. Therefore, older women were included in this study. The women sampled for this study lived in one of 17 localities in the Bogotá capital district. Individuals, families and even whole groups with different sociodemographic characteristics arrive in Bogotá from different regions, fleeing poverty, exile and displacement due to different types of violence: sociopolitical violence related to the armed conflict, violence related to drug trafficking, violence related to common crime or domestic violence. The levels of poverty, insecurity, profound inequality and social injustice are reproduced and reinforced at the mercy of a patriarchal culture that promotes gender inequality, subjugation, exclusion and victimization of women [49]. This is the context in which IPV against women occurs and is articulated with other types of violence, making it more complex to understand and prevent [50]. Violence against women is a common, widespread and repeated practice in Colombia [51]. Studies conducted in Colombia have found symptoms and/or disorders of depression, anxiety and post-traumatic stress among women exposed to IPV [52,53,54]. In a study by Molina et al. [55] on psychopathology and IPV against women during the COVID 19 pandemic, the most common symptoms found were somatization, depression, psychoticism and phobic anxiety, with percentages ranging from 40.6% to 32.2%. Between 29.3% and 24.1% of women had symptoms of anxiety, obsessive-compulsive, interpersonal sensitivity, hostility and suicidal ideation, and 17% had global distress.

Based on previous research on the negative mental health consequences of IPV victimization for women, as well as the protective role that self-esteem and social support have on women’s health and well-being during times of stress, such as the recent COVID-19 pandemic [56], we propose the following hypotheses:

**Hypothesis** **1.** *Women exposed to more psychological, physical and sexual IPV experience more mental distress.*

**Hypothesis** **2.** *Women exposed to more psychological, physical and sexual IPV experience more post-traumatic stress symptoms*.

**Hypothesis** **3.** *Women exposed to more psychological, physical and sexual IPV experience lower life satisfaction.*

**Hypothesis** **4.** *Women with greater perceived social support experience less mental distress, fewer post-traumatic stress symptoms and greater life satisfaction.*

**Hypothesis** **5.** *Women with higher self-esteem experience less mental distress, fewer post-traumatic stress symptoms and greater life satisfaction.*

## 2. Materials and Methods

### 2.1. Procedure

The sample was recruited through centers for the care of battered women (family commissariats, hospitals and service providers), as well as work and educational centers and sports, cultural and social groups. The WHO Ethical and Safety Recommendations for Research on Domestic Violence against Women [57] were considered in the development of the research. This research is part of a larger study on IPV against women living in different geographical areas, corresponding to the analysis of women living in Colombia, and was approved by the Research Ethics and Animal Welfare Committee of the University of La Laguna. Contacts were made with the different institutions, centers and groups to request their collaboration and to be able to make the appropriate call. In all cases, the research was presented to the women. With those who agreed to collaborate, a schedule was established, and the conditions of the call and the application of the questionnaires were agreed upon, following the ethical principles established throughout the process. In exchange for their collaboration, some institutions requested a conference, the evaluation of an instrument or the commitment to present the results at the end of the research, activities that have already been carried out.

The questionnaires and assessment inventories were administered by a team consisting of three psychologists from the institutions that had agreed to participate in the study and three psychology students in their final semester of clinical psychology training who knew how to conduct a crisis intervention. All women were informed of the nature of the research, the completely voluntary nature of their participation, and the confidentiality of the data. Women who agreed to participate were first given the informed consent form to sign. They were then given the assessment instruments and instructions on how to complete them. During the completion of the tests, the research team was attentive to participants’ doubts. The average time to complete the questionnaires was 45 min.

### 2.2. Participants

The sample is non-probability and consists of 255 women aged 18 to 71 who reside in 17 different localities in the Bogotá capital district and who have or recently had a partner. The main sociodemographic characteristics of these women are presented in Table 1. As can be seen, there is diversity in their educational levels and socioeconomic status, although more than half (64.3%) had a low socioeconomic status, 26.7% had a medium-low socioeconomic status, and only two women had a medium-high socioeconomic status. Most of the women had a high school education (42.0%) or vocational training (26.3%), although almost a fifth (19.6%) had a university education. Although the age range of the women was wide, they were most often between 30 and 44 years old, which was the case for 42%, or between 20 and 29 years old, which was the case for just over a third. Older women were the least common, with only 4.3% aged 60 or older.

The most common marital status was living with a partner (47.8%). Fifty-three women (20.8%) were married, 9.0% had never been married, four women were separated, divorced or widowed, and 20.8% were in the process of separation.

### 2.3. Instruments

#### 2.3.1. Inventory for the Assessment of Partner Abuse of Women (PAWI) [58]

The PAWI assesses IPV against women in terms of the three main types of violence against women: physical violence, psychological violence and sexual violence. The response scale has five alternatives: never (score 0), sometimes (score 1), half the time (score 2), often (score 3) and almost always (score 4). The inventory consists of 57 items grouped into three scales: (1) psychological violence, consisting of 38 items that include behaviors such as insulting, belittling, threatening, humiliating, blaming or controlling; (2) physical violence, consisting of 15 items that include behaviors such as pushing, slapping, throwing objects, pulling hair, hitting, threatening with knives or sharp objects, biting and choking; (3) sexual violence, consisting of 4 items. The PAWI has been used in studies carried out in Spain [34] and in some Latin American countries [38,59], which have shown that the internal consistency is higher than 0.85.

#### 2.3.2. The Spanish Version of the Goldberg General Health Questionnaire (GHQ-28) [60]

The Goldberg General Health Questionnaire is an instrument designed to detect psychological disorders in community and non-psychiatric clinical settings, focusing on the actual psychological components of illness and referring to two main types of phenomena: the inability to continue performing normal health functions and the occurrence of phenomena of mental distress [61]. The GHQ-28 consists of 28 items organized into four subscales: somatic symptoms, anxiety and insomnia, social dysfunction and severe depression. The GHQ allows for different scoring methods, and the present study used the Likert method with weights ranging from 0 to 3, with higher scores indicating greater mental distress. To obtain a measure of mental distress, the total score was used in the present study, where the scores of the 28 items were summed.

#### 2.3.3. Post-Traumatic Stress Disorder Symptom Severity Scale [62]

This is a scale that measures post-traumatic stress. It is based on the DSM-IV diagnostic criteria. The scale is designed to provide a categorical diagnosis of the disorder and to assess its severity by quantifying both the frequency and intensity of symptoms. It consists of a total of 17 items, with 5 items assessing re-experiencing, 7 assessing avoidance and 5 assessing increased arousal. In the present study, the total score of the 17 items comprising the scale was used.

#### 2.3.4. Self-Esteem Inventory (SEQ-MR)

The SEQ-MR is a self-esteem assessment instrument. It is the Spanish version of the Self-esteem Questionnaire, reduced and validated for battered women [63]. It consists of 25 items with a four-point response scale ranging from 0 (never) to 3 (always). Higher scores indicate higher self-esteem.

#### 2.3.5. Social Support Scale [64]

The Social Support Scale measures functional social support by assessing the extent to which people feel they have both emotional, informational and instrumental social support. It consists of 12 items with a four-point response format ranging from 0 (never) to 3 (always). Higher scores indicate greater perceived social support.

#### 2.3.6. Satisfaction with Life Scale (SWLS) [65]

The SWLS measures an individual’s overall assessment of life satisfaction and consists of 5 items. The response format is a 7-point Likert scale ranging from strongly disagree (score 1) to strongly agree (score 7). Higher scores indicate greater life satisfaction.

#### 2.3.7. Sociodemographic Characteristics Data Sheet

This sheet collects the main sociodemographic characteristics of the women, including age, education and socioeconomic status. The socioeconomic status is based on the socioeconomic stratification, a classification of housing that is made according to the Regime of Domiciliary Public Services in Colombia (Law 142 of 1994) [66]. The Socioeconomic Stratification is divided into 6 levels: Low-Low, Low, Middle-Low, Middle, Middle-High and High.

### 2.4. Data Analysis

Cronbach’s Alpha was used to examine the internal consistency of the measured factors. Correlational analyses were carried out using Pearson’s correlation coefficient, except for socioeconomic status and education, which were calculated using Spearman’s Rho, as these variables are ordinal. To determine the relevance of the different types of violence (physical, psychological and sexual) on women’s mental distress, post-traumatic stress symptoms and life satisfaction, as well as the relevance of sociodemographic characteristics, self-esteem and social support in this association, a hierarchical multiple regression analysis was performed. As the first three hypotheses of the study proposed that women exposed to more psychological, physical and sexual IPV experience more mental distress, more post-traumatic stress symptoms and less life satisfaction, only the three types of IPV were included in the first step (Model 1). In the second step (Model 2), women’s age, education and socioeconomic status were added to discover whether these variables could improve the prediction of psychological distress, post-traumatic stress symptoms and life satisfaction. Finally, in the third step (Model 3), self-esteem and social support were added.

## 3. Results

Table 2 shows the descriptive statistics (mean, standard deviation, median, minimum, maximum, skewness and kurtosis) and the internal consistency of the study variables. As can be seen, the internal consistency of most of the variables was excellent, exceeding the value of 0.90, and for two of the variables (life satisfaction and sexual IPV) it was good, with values of 0.89 and 0.87, respectively. The analysis of IPV reported by women revealed a wide range. Scores on the total IPV ranged from 11 to 225, with the maximum score allowed by the inventory being 228; the mean score was 99.79, the standard deviation (*SD*) was 49.92, and the median was 106. Scores for psychological IPV ranged from 9 to 149, with the maximum score allowed by the scale being 152; the mean score was 77.92, the *SD* was 35.91 and the median was 88. Physical IPV scores ranged from 0, which occurred in 11.4%, to 60, the maximum score allowed by the scale, which occurred in two of the women; the mean score was 18.05, the *SD* was 14.52, and the median was 15. Sexual IPV scores ranged from 0, which occurred in 35.3%, to 16, the maximum score allowed by the scale, which occurred in 5 of the women; the mean score was 3.82, the *SD* was 4.34, and the median was 2.

Table 3 shows the Pearson correlations between the study variables. The three types of IPV were statistically significantly and positively correlated. Exposure to IPV was correlated with all study variables except women’s age, and sexual IPV was not statistically significantly correlated with socioeconomic status. In addition, women’s age was not statistically significantly correlated with their self-esteem, mental distress, social support and life satisfaction. Socioeconomic status was not correlated with women’s self-esteem. Post-traumatic stress symptoms and mental distress were strongly correlated (*r* = 0.76, *p* < 0.001), and both variables were negatively correlated with life satisfaction.

Table 4 shows the main results of the hierarchical multiple regression analysis predicting mental distress. Psychological and sexual IPV were statistically significant in the first step (Model 1), although the beta weight of psychological violence was higher than that of sexual violence. This model accounted for 28% of the mental distress scores. Adding sociodemographic variables in Model 2 did not produce a significant change in *R*^2^, whereas adding self-esteem and social support in Model 3 produced a significant increase in *R*^2^ (*R*^2^ change = 0.22). In the final model, with all variables in the regression equation, only three were statistically significant: psychological IPV, self-esteem and social support, with greater mental distress for women with lower self-esteem, those who had been exposed to greater psychological IPV, and those who perceived less social support. The total accounted variance in distress scores was 50%.

Table 5 shows the main results of the regression analysis predicting post-traumatic stress symptoms. As can be seen, all three models were statistically significant, although the change in *R*^2^ in the second step (Model 2), where the sociodemographic variables were added, was only 0.03. In the first step (Model 1), only psychological and sexual IPV were statistically significant, with the beta weight of psychological violence being much higher than that of sexual violence. This model accounted for 30% of the variance in post-traumatic stress symptoms. Of the sociodemographic variables included in the second step, only age was statistically significant, while of the two variables added in the third step (self-esteem and social support), only self-esteem was statistically significant, and the amount of variance explained increased (*R*^2^ change = 0.14). In the final model, with all variables in the regression equation, only three of the variables were statistically significant, and the variable with the greatest predictive power for post-traumatic stress symptoms was psychological IPV, followed by self-esteem and women’s age. Women with more post-traumatic stress symptoms were those exposed to more psychological IPV, those with lower self-esteem and those who were younger. The total percentage of predicted variance in post-traumatic stress symptoms was 46%.

The main results of the regression analysis predicting life satisfaction are presented in Table 6. As can be seen, all three models were statistically significant, although the change in *R*^2^ was much smaller in the second step (Model 2) in which the sociodemographic variables were included (*R*^2^ change = 0.04). Model 1 accounted for 32% of the variation in life satisfaction, and all three types of IPV had statistical significance, but the addition of sociodemographic characteristics in Model 2 removed physical IPV from significance. Only psychological IPV remained significantly associated with higher life satisfaction when self-esteem and social support were added to the regression equation in Model 3. In this model, with all variables included in the regression equation, five of the variables were statistically significant. Women with higher life satisfaction were those who had higher self-esteem, higher social support, less exposure to psychological IPV, were older and had more education. This model accounted for 58% of the variance in life satisfaction scores.

## 4. Discussion

This study examined the associations between IPV against women and mental distress, post-traumatic stress symptoms and life satisfaction in a sample of Colombian women. It also analyzed whether age, higher socioeconomic status and educational attainment, as well as higher self-esteem and social support, are associated with better mental health and well-being. Results showed that although there was great variability in the frequency and severity of IPV to which they had been exposed, all women reported experiencing psychological IPV. Most women (88.6%) had also experienced physical IPV, and more than half (64.5%) reported exposure to sexual IPV. These findings are consistent with those from other studies in which psychological violence was the most prevalent form of IPV [7,11,37,67].

We hypothesized (Hypothesis 1) that women exposed to more psychological, physical, and sexual IPV experience more mental distress. Results showed that sexual and psychological IPV were predictive of mental distress, but physical IPV was not. In addition, the association between sexual violence and mental distress was no longer statistically significant when women’s self-esteem and social support were added to the regression equation. Thus, Hypothesis 1 was only partially supported. When post-traumatic stress symptoms were considered as the dependent variable, a similar phenomenon occurred: the beta weights of psychological and sexual violence were statistically significant in Model 1, which included only the three types of IPV, and in Model 2, which added women’s age, education, and socioeconomic status. But in Model 3, the inclusion of self-esteem and social support in the regression equation decreased the beta value of sexual IPV and it lost statistical significance. In the final model, of the three types of IPV, only psychological IPV was statistically significant in predicting women’s post-traumatic stress symptoms. However, psychological IPV was the most important predictor of such symptoms. Therefore, Hypothesis 2, which stated that women exposed to more psychological, physical, and sexual IPV experience more post-traumatic stress symptoms, was only partially supported.

Hypothesis 3 proposed that women exposed to more psychological, physical, and sexual IPV experience lower life satisfaction. All three types of IPV examined, psychological, physical and sexual, were significant predictors in Model 1 of the hierarchical regression analysis with life satisfaction as the dependent variable. An adjusted *R*^2^ of 0.32 indicated that nearly one third of variability in women’s life satisfaction was predicted by IPV, with greater life satisfaction among women who experienced less psychological, physical and sexual IPV. The beta value for physical violence was no longer statistically significant when women’s age, education and socioeconomic status were added to the equation in Model 2. And in Model 3, only psychological violence remained statistically significant when self-esteem and social support were added to the regression equation, with lower life satisfaction among women who experienced more psychological IPV. Therefore, Hypothesis 3 was only partially supported.

The fourth hypothesis proposed that women with greater perceived social support experience less mental distress, fewer post-traumatic stress symptoms and greater life satisfaction. Correlational analyses revealed statistically significant and negative associations between perceived social support and life satisfaction, mental distress and post-traumatic stress symptoms, although the magnitude of the correlation coefficient between perceived social support and post-traumatic stress symptoms was smaller than for the other two variables, sharing only 13% of the variance. Regression analyses indicated that social support was a statistically significant predictor of women’s mental distress and life satisfaction, with lower mental distress and higher life satisfaction among women with higher perceived social support. However, social support was not a statistically significant predictor of post-traumatic stress symptoms, and the beta weight was of only −0.09. Hypothesis 4 was therefore not fully supported.

The fifth hypothesis proposed that women with higher self-esteem experience less mental distress, fewer post-traumatic stress symptoms and greater life satisfaction. Regression analyses indicated that self-esteem was the most important predictor of women’s mental distress and life satisfaction, and the second most important factor in predicting post-traumatic stress symptoms, with greater mental distress and post-traumatic stress symptoms and less life satisfaction in women with lower self-esteem. Hypothesis 5 was therefore fully supported.

Taken together, these findings suggest that IPV poses a significant threat to women’s mental health, which is consistent with previous research [11,21,22,24,25,26,30,31,32,33,34,35,36,37,38,39,40,41,42,51,52,53,54,55]. Furthermore, the results of the present study show that of the three types of IPV examined (physical, psychological and sexual), psychological IPV is associated with greater psychological harm and that the effects of such violence appear to be more difficult to counteract by personal factors, such as women’s high self-esteem, or by social factors, such as high perceived social support. The importance of psychological violence for the mental health of the victim has also been found in previous studies [7,9,18,33,35,37,39,67,68], and the results of the present study support the approach of Heise et al. [7] (p. 1) that psychological violence is “linked to negative health outcomes among women and is frequently identified as more wounding than physical or sexual violence”. Of the three types of IPV against women analysed in this study, the type of IPV that proved least relevant in predicting women’s mental health symptoms and well-being was physical IPV. Although physical IPV was statistically significantly and positively associated with greater mental distress, more post-traumatic stress symptoms and lower life satisfaction in the bivariate analyses, it was not a statistically significant predictor of these outcomes in the regression analyses and only predicted lower life satisfaction in the model including only the three types of IPV. In any case, it is important to note that most of the women in the sample (88.6%) had also experienced physical violence, so the effect of physical violence on women’s mental distress and post-traumatic stress symptoms was only statistically controlled for. In addition, the data from this study showed that physical and psychological violence were closely linked.

The results of the present study also showed that more IPV was associated with lower life satisfaction, findings that are consistent with those of other studies [69,70]. Notably, in the present study, the three types of IPV (physical, psychological and sexual) predicted almost one-third of the variance in life satisfaction, which was higher among women who reported less IPV. Although such an association was found in the bivariate correlations and regression analysis in Model 1, which included only the three types of violence, when age, socioeconomic status and women’s education were added in Model 2, the beta weight of physical violence decreased to −0.14 and was no longer statistically significant. Of the three variables added in Model 2, two were statistically significant: age and education level, with more educated and older women being more satisfied with life. And when self-esteem and social support were added to the regression equation in Model 3, the beta weight of sexual violence dropped to −0.11 and was no longer statistically significant. Although both variables were statistically significant, with higher life satisfaction among women with higher self-esteem and social support, self-esteem was shown to be more relevant to life satisfaction than social support, as the magnitude of its beta weight was much higher. However, although the beta weight of psychological IPV decreased slightly in Model 2 (from −0.25 to −0.21) and in Model 3 to −0.16, it remained statistically significant in all three models. This suggests that the effect of physical IPV on women’s life satisfaction may be mediated or moderated by sociodemographic characteristics and that of sexual violence by self-esteem and social support. However, these moderating and/or mediating effects appear to be more limited in the case of psychological IPV. These are hypotheses to be tested in future work.

The positive association of social support with the health and well-being of women exposed to IPV found in the present study is consistent with findings from previous research [36,71]. Although greater social support was associated with fewer post-traumatic stress symptoms in bivariate analyses in the present study, social support was not a statistically significant predictor of post-traumatic stress symptoms in regression analysis. It is also interesting to note that, as mentioned above, psychological IPV was the main predictor of such symptoms. These are interesting findings, the causes of which are unknown and should be addressed in future work. In any case, the relevance of the contextual factors of the country in which the study was conducted, Colombia, a country that has experienced decades of armed conflict, cannot be ruled out. This armed conflict has had a significant impact on citizens and has also led to a huge displacement of the population from the countryside to the cities after experiencing or witnessing much violence, creating complex social, economic, political and cultural problems, including the normalization of IPV [72].

There are several limitations to the present study. First, it is a cross-sectional study, which means that causal relationships cannot be established. Second, all measures are self-reported, which may introduce biases, including recall bias and social desirability bias. Third, the sample is non-probabilistic and all women resided in the capital district of Bogotá, which should be taken into account when generalizing the results. In addition, although the age range of the participating women was very broad, there were few older women, with only 4.3% aged 60 or older. Finally, the present study focused on a general analysis of the association between IPV against women and women’s mental health and well-being, without a specific study or a study based on a person-centered approach.

## 5. Conclusions

IPV is a major threat to women’s mental health and well-being. Although high self-esteem is the most important predictor of lower mental distress and higher life satisfaction among women exposed to IPV, the same is not true for post-traumatic stress symptoms, for which higher psychological IPV is the most important predictor, although lower self-esteem and women’s younger age are also associated with greater post-traumatic stress symptoms. Psychological violence is also an important predictor of mental distress, with greater mental distress among women exposed to greater psychological violence; greater psychological violence is also associated with lower life satisfaction. Greater social support is also associated with greater life satisfaction and lower mental distress among women exposed to IPV. The findings of this study have practical implications and are relevant to policies, strategies and programs of psychological intervention aimed at the eradication of violence against women and the recovery of its victims.

## Figures and Tables

**Table 1:** Sociodemographic characteristics of women participants.

Characteristic	*n*	%
Education		
No schooling	1	0.4
Primary school	30	11.8
Secondary school	107	42.0
Vocational training	67	26.3
University degree	40	15.7
Postgraduate	10	3.9
Age ranges		
18–19	16	6.3
20–29	88	34.5
30–44	108	42.4
45–59	32	12.5
60–71	11	4.3
Socioeconomic status		
Low-low	16	6.3
Low	164	64.3
Middle-low	68	26.7
Middle	5	2.0
Middle-high	2	0.8
Marital status		
Never married	23	9.0
Married	53	20.8
Living with a partner	122	47.8
Separation process	53	20.8
Separated/divorced/widowed	4	1.6

**Table 2:** Descriptive statistics of study variables.

Variable	Mean	*SD*	Mdn.	Min.	Max.	Skew.	Kurt.	Alpha
Psychological IPV	77.92	35.91	88	9	149	−0.32	−1.02	0.96
Physical IPV	18.05	14.52	15	0	60	0.49	−0.71	0.93
Sexual IPV	3.82	4.34	2	0	16	1.03	0.14	0.87
Total IPV	99.79	49.92	106	11	225	−0.18	−0.97	0.97
Self-esteem	46.05	17.32	49	2	75	−0.42	−0.69	0.96
Social support	19.21	10.02	18	0	36	0.05	−1.06	0.92
Mental distress	32.96	16.01	32	3	77	0.32	−0.81	0.94
Post-traumatic stress symptoms	20.25	13.73	19	0	49	0.19	−1.06	0.95
Life satisfaction	17.81	8.12	17	5	35	0.24	−1.02	0.89

**Table 3:** Pearson correlation coefficients between the study variables.

Variable	1	2	3	4	5	6	7	8	9	10
1. Psychological IPV										
2. Physical IPV	0.69 ***									
3. Sexual IPV	0.55 ***	0.66 ***								
4. Women’s age	−0.11	−0.06	0.10							
5. Education ^&^	−0.24 ***	−0.32 ***	−0.21 **	−0.16 *						
6. Socioeconomic status ^&^	−0.17 **	−0.21 **	−0.12	0.19 **	0.38 ***					
7. Self-esteem	−0.32 ***	−0.37 ***	−0.36 ***	0.00	0.26 ***	0.09				
8. Social support	−0.29 ***	−0.26 ***	−0.27 ***	−0.02	0.19 **	0.14 *	0.41 ***			
9. Mental distress	0.51 ***	0.41 ***	0.43 ***	−0.11	−0.14 *	−0.15 *	−0.61 ***	−0.43 ***		
10. Post-traumatic stress symptoms	0.55 ***	0.39 ***	0.39 ***	−0.18 **	−0.15 *	−0.15 *	−0.54 ***	−0.36 ***	0.76 ***	
11. Life satisfaction	−0.50 ***	−0.52 ***	−0.47 ***	0.12	0.29 ***	0.17 **	0.66 ***	0.47 ***	−0.65 ***	−0.57 ***

Note. & = Spearman’s Rho correlations. * *p* < 0.05; ** *p* < 0.01; *** *p* < 0.001.

**Table 4:** Summary of the hierarchical regression with mental distress as the dependent variable.

Variable	Model 1			Model 2			Model 3		
	B(*SE*)	β	*t*-Value	B(*SE*)	β	*t*-Value	B(*SE*)	β	*t*-Value
Psychological IPV	0.17(0.03)	0.37	4.81 ***	0.15(0.04)	0.35	4.38 ***	0.13(0.03)	0.30	4.55 ***
Physical IPV	0.01(0.09)	0.01	0.15	−0.00(0.10)	−0.00	−0.03	−0.06(0.08)	−0.06	−0.75
Sexual IPV	0.78(0.27)	0.22	2.91 **	0.90(0.28)	0.25	3.27 **	0.43(0.23)	0.12	1.82
Women’s age				−0.14(0.08)	−0.10	−1.69	−0.10(0.07)	−0.07	−1.40
Education				−0.04(51)	−0.01	−0.08	0.79(0.43)	0.10	1.81
Socioeconomic status				−0.80(1.57)	−0.03	−0.51	−1.32(1.31)	−0.05	−1.01
Self-esteem							−0.41(0.05)	−0.44	−8.27 ***
Social support							−0.26(0.08)	−0.16	−3.18 **
*R* ^2^	0.29			0.30			0.51		
Adjusted *R*^2^	0.28			0.28			0.50		
*R*^2^ Change	0.29 ***			0.01			0.22 ***		

Note. B (β) = unstandardized (standardized) regression coefficient. *R*^2^ = explained variance. ** *p* < 0.001; *** *p* < 0.001.

**Table 5:** Summary of the hierarchical regression with the post-traumatic stress symptoms as the dependent variable.

Variable	Model 1			Model 2			Model 3		
	B(*SE*)	β	*t*-Value	B(*SE*)	β	*t*-Value	B(*SE*)	β	*t*-Value
Psychological IPV	0.19(0.03)	0.50	6.62 ***	0.18(0.03)	0.46	6.07 ***	0.16(0.03)	0.43	6.28 ***
Physical IPV	−0.05(0.08)	−0.06	−0.69	−0.09(0.08)	−0.09	−1.11	−0.14(0.07)	−0.15	−1.97
Sexual IPV	0.48(0.22)	0.15	2.13 *	0.64(0.23)	0.21	2.82 **	0.33(0.21)	0.11	1.60
Women’s age				−0.21(0.07)	−0.18	−3.08 **	−0.18(0.06)	−0.16	−2.99 **
Education				−0.50(0.43)	−0.08	−1.15	0.08(0.39)	0.01	0.21
Socioeconomic status				0.47(1.31)	0.02	0.36	−0.03(1.17)	−0.00	−0.02
Self-esteem							−0.30(0.04)	−0.38	−6.87 ***
Social support							−0.12(0.07)	−0.09	−1.70
*R* ^2^	0.31			0.34			0.48		
Adjusted *R*^2^	0.30			0.32			0.46		
*R*^2^ Change	0.31 ***			0.03 *			0.14 ***		

Note. B (β) = unstandardized (standardized) regression coefficient. *R*^2^ = explained variance. * *p* < 0.05; ** *p* < 0.001; *** *p* < 0.001.

**Table 6:** Summary of the hierarchical regression with life satisfaction as the dependent variable.

Variable	Model 1			Model 2			Model 3		
	B(*SE*)	β	*t*-Value	B(*SE*)	β	*t*-Value	B(*SE*)	β	*t*-Value
Psychological IPV	−0.06(0.02)	−0.25	−3.33 **	−0.05(0.02)	−0.21	−2.78 **	−0.04(0.01)	−0.16	−2.62 **
Physical IPV	−0.12(0.05)	−0.21	−2.57 *	−0.09(0.05)	−0.14	−1.69	−0.05(0.04)	−0.08	−1.19
Sexual IPV	−0.37(0.13)	−0.20	−2.80 **	−0.45(0.13)	−0.24	−3.83 **	−0.21(0.11)	−0.11	−1.94
Women’s age				0.10(0.04)	0.15	2.63 **	0.09(0.03)	0.12	2.68 **
Educational level				0.87(0.25)	0.22	3.55 ***	0.43(0.20)	0.11	2.13 *
Socioeconomic status				−0.62(0.75)	−0.05	−0.82	−0.27(0.61)	−0.02	−0.43
Self-esteem							0.21(0.02)	0.43	8.83 ***
Social support							0.14(0.04)	0.18	3.75 ***
*R* ^2^	0.33			0.37			0.59		
Adjusted *R*^2^	0.32			0.35			0.58		
*R*^2^ Change	0.33 ***			0.04 **			0.22 ***		

Note. B (β) = unstandardized (standardized) regression coefficient. *R*^2^ = explained variance. * *p* < 0.05; ** *p* < 0.001; *** *p* < 0.001.

## Data Availability

Data are unavailable due to ethical restrictions.
